# Early detection of mastitis in cows using the system based on 3D motions detectors

**DOI:** 10.1038/s41598-022-25275-2

**Published:** 2022-12-08

**Authors:** Grzegorz Grodkowski, Tomasz Szwaczkowski, Krzysztof Koszela, Wojciech Mueller, Kamila Tomaszyk, Ton Baars, Tomasz Sakowski

**Affiliations:** 1 Department of Animal Breeding, Institute of Animal Sciences, Warsaw University of Life Sciences, Warsaw, Poland; 2 Department of Genetics and Animal Breeding, Poznan University of Life Sciences, Poznan, Poland; 3 Department of Biosystems Engineering, Poznań University of Life Sciences, Poznan, Poland; 4 Department of Mathematical and Statistical Methods, Poznań University of Life Sciences, Poznan, Poland; 5 Department of Immunopharmacology, Utrecht University, Utrecht, The Netherlands; 6 Department of Biotechnology and Nutrigenomics, Institute of Genetics and Animal Biotechnology, Jatrzębiec, Poland

**Keywords:** Diseases, Health care, Engineering

## Abstract

Mastitis is one of the major health problems in dairy herds leading to a reduction in the leading to a reduction in the quality of milk and economic losses. The research aimed to present the system, which uses electronic 3D motion detectors to detect the early symptoms of mastitis. The system would allow more effective prevention of this illness. The experiment was carried out on 118 cows (64 Holstein Friesian and 54 Brown Swiss). The animals were kept in free-stall barn with access to pasture. The occurrence of mastitis cases was noticed in veterinary register. Microbiological culture was taken from milk in order to confirm the development of infection. Data from motion detectors were defined as time spent by animals on feed intake, ruminating, physical activity and rest, and were expanded by adding information about feeding group, breed type and lactation number. During analyses, two approaches were used to process the same dataset: artificial neural networks (ANN) and logistic regression. The obtained ANN and the logistic regression models proved to be satisfactory from the perspective of applied criteria of goodness of fit (area under curve—exceed 0.8). Quality parameters (accuracy, sensitivity and specifity) of logistic regression are relatively high (larger than 0.73), whereas the ranks of significance of the studied variables varied across datasets. These proposed models can be useful for automating the detection of mastitis once integrated into the farm’s IT system.

## Introduction

Mastitis is one of the most common conditions which affect herds of dairy cattle. The occurrence of undiagnosed cases of mastitis in herd leads to an increased average of somatic cells content in milk, which worsens its technological properties e.g. reduced thermostability of milk or reduced cheese yield. Mastitis milk has negative effect on milk-processing because of increased enzymatic activity, the effect of which is lower efficiency of butter and cheese. As Halasa et al.^1^ reported this condition also causes an increase in cost of treatment and in labour cost. Average cost of treatment of a single cow affected by mastitis in Western Europe is about Euro 27–43. After including all costs associated with mastitis such as: losses in milk production, costs of treatment, possible slaughter and other complications, yearly cost per cow is—according to various authors—is from USD 71^2^ to USD 435^3^. By reason of increased breeding intensity one can also observe higher and higher costs connected with mastitis resulting from, among other things, early removal of animals from the herd. It also should be highlighted that mastitis has negative impact on cattle well-being^4^. It is observed that cows affected by this condition are characterized by lower motion activity, lower feed consumption and social behaviour disorders^5^. That is why early diagnosis of mastitis can lead to cost reduction and improvement of animal well-being. Moreover, increasing number of large dairy farms hat that is observable both in the USA and Europe makes breeders invest in automated production solutions.

At present two methods are used to detect mastitis, namely specific electrical conductivity^6^ and content of somatic cells in milk^7^, although it is very often diagnosed by visual confirmation of clinical signs. Devices that are used to detect mastitis are attached to teat cups or are embedded in interceptors in milking halls. On the other hand, the sensor system can be perceived as valuable alternative or extension of production automation to help with of mastitis detection. It causes additional increase in costs and complications in barn fittings. In recent years there has been a rapid development of information and measurement systems, which use various types of motion detectors in order to support identification of behavioural patterns^8^. The above solutions serve to detect heat stress^9^, lameness^10^ and monitor time of ruminating^11^. Apart from the above, one can observe the use of systems based on other types of microcomputers, which allow to monitor pH in rumen, which is a good sign of problems with metabolism^12,13^. Other independent solution is the system of detecting mastitis during milking process^4^, to which is included, among other things, advanced robotic milking. The aforementioned inflammatory condition of a cow’s udder has a substantial influence on a cow’s activity, which is also confirmed by other authors^4,5,14^. That is why it seems justified to claim that monitoring of cows’ behaviour in herd can also serve as a diagnostic sign of mastitis. At present there are systems equipped with electronic motion detectors, which allow to recognize various forms of cows’ behaviours. After appropriate processing and after building proper models, information coming from such detectors can be the basis for early prognosis of mastitis occurrence. Nowadays, a number of mathematical approaches are known, which allow to predict of the value of dependent binary variable, starting from regression methods^15^, through stochastic modelling^16^ and modelling with Artificial Neural Networks (ANN)^17^.

The applied logistic regression model not only allows to determine if change in cow’s behaviour had a substantial impact on pronouncing mastitis but also it will allow to identify which of the variables can be prognosis variables, which can be used to assess probability of mastitis development. An alternative approach to the signalled issue is the use of ANN, which is one of the methods of artificial intelligence. In recent years ANN has become popular to such an extent that now it is difficult to find a field of science, in which there were no endeavours made to make use of the effectiveness of this tool. ANN allow filtration of signals, elimination of noises, classification of images and steering robots. ANN is an effective tools, which allows reproducing complicated between properly selected input variables, and properly defined output variables. In cases with classification problems during generating models (testing, validation, verification), the factors which have impact on the quality of parameters in a network are: speed of learning, size of errors and generalization capability.

The main aim of the research was to verify the possibilities of building up classification models with statistical tools and methods of artificial intelligence based on data obtained from the systems based on 3D motion detectors. The above model would make it possible to detect health conditions such as mastitis at an early stage. Moreover, due to the increasing number of large dairy farms in both the USA and Europe, breeders are more likely to invest in automated production solutions.

## Results

### Neural modeling

The built of neural models as well as estimation of parameters concerning logistic regression models went according to the rule of top-down approach. It means that while creating both types of models, data coming from the biggest HF-BS set, were taken. The most optimal network for the aforementioned HF-BS set was neural typology type: 16–12-1 (Multilayer Perceptron) including 16 neurons in the input layer, 12 neurons in the hidden layer and 1 neuron in the output layer. Values of individual errors obtained for this model; learning, validation, testing and quality parameters corresponding to them in combination with AUC measurement were presented in Table 1.

**Table 1:** List of created neural models, error values, accuracy and AUC for individual sets

Data set/count	Model	Learning error	Val. error	tst error	Learning quality	Validation quality	Testing quality	AUC
HF-BS/3735	MLP 16:12:1	0.1799	0.2051	0.2044	0.7698	0.7762	0.7878	0.8433
HF/1989	MLP 15:19:9:1	0.1975	0.2294	0.2211	0.8010	0.8149	0.8008	0.8593
BS/1746	MLP 15:12:8:1	0.1633	0.1715	0.1670	0.7595	0.8169	0.8165	0.8255
HF-CO/1224	MLP 14:9:6:1	0.2345	0.2310	0.2653	0.7582	0.7516	0.7876	0.8645
HF-PA/765	MLP 14:4:1	0.1735	0.2027	0.2026	0.7963	0.7801	0.7644	0.8793
BS-CO/1032	MLP 14:5:1	0.1443	0.1057	0.1496	0.7539	0.7364	0.7907	0.8012
BS-PA/714	MLP 14:4:1	0.1169	0.2231	0.1499	0.7675	0.7933	0.7921	0.8569

*MLP *multi layer perceptron, *AUC* area under curve.

In Table 1, characteristics of individual models were put together, namely the number of input neurons, the number of neurons in the hidden layer and the number of neurons in the output layer.

MLP neural models that were created are characterized by similar error values (of course slight differences can be noticed) from the perspective of classification of animals suffering from mastitis. Similar observations were made on the basis of AUC determining the surface area under ROC curse (Table 2). Creating models based on Statistica involved doing analysis of sensitivity of the obtained networks. Ranks of quantitative and qualitative variables together with their error quotient for individual MLP neural models were presented in Table 2. The sensitivity analysis is presented separately for the learning and validation set (Table 2). These indications are a valuable indicator of the correctness of a given assessment. The sensitivity is presented in the form of error ratio and rank:

- error ratio—it is a ratio of error to error obtained using all the independent characteristics; the greater the ratio, the greater the significance of given characteristics;
- rank—indicator characterizing numerical characteristics in order of decreasing error, where a rank of 1 is the most important for the network.

**Table 2:** Ranks of variables with error quotient for seven neural models.

Data set Rank/error quotient	HF-BS	HF	BS	HF-CO	HF-PA	BS-CO	BS-PA
AV-TACTIV	**5/1.0750**	**5/1.0878**	10/1.0025	11/1.0044	7/1.0030	**3/1.0882**	7/1.1139
SD-TACTIV	14/1.0118	10/1.0379	14/0.9999	15/0.9997	6/1.0036	14/1.0020	**4/1.2963**
AV-ACTIV	9/1.0321	13/1.0233	12/1.0018	**4/1.0342**	15/0.9988	8/1.0290	14/0.9994
SD-ACTIV	8/1.0517	15/1.0042	15/0.9994	12/1.0024	13/0.9998	7/1.0343	13/1.0037
AV-HACTIV	15/1.0075	14/1.0098	**4/1.0257**	10/1.0048	**3/1.0081**	12/1.0102	**5/1.2174**
SD-HACTIV	11/1.0226	8/1.0534	8/1.0086	13/1.0006	11/1.0017	**4/1.0754**	**3/1.3453**
AV-INTAKE	13/1.0163	7/1.0609	13/1.0008	6/1.0173	**5/1.0045**	**2/1.1757**	11/1.0080
SD-INTAKE	**2/1.1185**	**2/1.1832**	**1/1.1250**	**5/1.0200**	9/1.0019	9/1.0237	**1/2.0641**
AV-RUMIN	16/1.0069	11/1.0376	7/1.0090	9/1.0060	14/0.9996	13/1.0036	9/1.0223
SD-RUMIN	10/1.0254	9/1.0532	11/1.0020	7/1.0119	8/1.0027	**5/1.0741**	8/1.0660
AV-REST	12/1.0201	12/1.0363	6/1.0098	8/1.0101	10/1.0018	11/1.0199	6/1.1902
SD-REST	6/1.0750	6/1.0628	**5/1.0109**	**3/1.0567**	**2/1.0149**	10/1.0208	12/1.0044
LOCAL	**3/1.1168**	**3/1.1605**	**3/1.0438**				
FEEDGR	**1/1.2456**	**1/1.1921**	**2/1.0584**	**1/1.1289**	**1/1.0201**	**1/2.8434**	**2/1.7219**
BREED	**4/1.1109**						
LACT	7/1.0671	**4/1.1251**	9/1.0059	**2/1.0749**	**4/1.0058**	6/1.0651	10/1.0133

*AV-TACTIV *average of total activity, *SD-TACTIV *standard deviation of total activity, *AV-ACTIV *average of activity, *SD-ACTIV *standard deviation of activity, *AV-HACTIV *average of high activity, *SD-HACTIV *standard deviation of high activity, *AV-INTAKE *average of feed intake, *SD-INTAKE* standard deviation of feed intake, *AV-RUMIN* average of rumination, *SD-RUMIN* standard deviation of rumination, *AV-REST* average of rest, *SD-REST *standard deviation of rest, *LOCAL* localization, *FEEDGR* feeding group, *BREED* breed, *LACT* lactation number (primiparous vs older cows).

Significant values are in bold.

### Logistic regression

The preliminary analysis (including estimation of correlation coefficients) was performed. In the process of selecting clarifying variables for logistic model within the frame of initial analysis, a correlation matrix was determined for continuous variables related to activity. For example in HF-BS set the highest positive correlation occurred between accumulative activity and activity (0.86), and negative correlation between time of taking feed and resting (−0.73) and between activity and ruminating (−0.54). Accumulative activity was also highly correlated with high activity (0.59) and ruminating (−0.54). Similar relations occurred in the remaining data subsets that were analysed. On account of correlations and occurrence co-linearity, accumulative activity was removed on this stage (Table 3). In case of HF-BS set in single analyses, where influence of each clarifying variable on logarithm of chance was researched, all variables with the exception of location had substantial influence on dependent variable. However, in multi-variable model, effect of location was significant. The tests that were done, which checked the linear relationship of logarithm of odds and independent variables, indicated curvilinear relationship in some cases. The above fact was the reason for adding variables of their squares to the set, which often resulted in elimination of variable itself, which was deemed unimportant and is presented in Table 3. Implementation of those variables substantially improved model matching. Estimation of logistic regression parameters for the seven datasets together with the importance of individual variables is presented in Table 3. However, measurements of matching quality and prediction, which show the adequacy of the created models, are presented in Table 4.

**Table 3:** Estimates of parameters of logistic regression equations.

Variable	HF-BS	HF	BS	HF-CO	HF-PA	BS-CO	BS-PA
AV-ACTIV	−0.200**	−0.295**					
(AV-ACTIV)^2^			−0.052**				0.312**
AV-HACTIV	−0.443**		−1.667**			−1.093**	3.542**
(AV-HACTIV)^2^		−0.037**					
AV-INTAKE	−0.496**	−0.790**	−1.029**		−0.579**	−0.281**	2.996**
(AV-INTAKE)^2^	0.009**	0.018**			0.015**		
AV-RUMIN				0.362**			2.499*
AV-REST			−1.224**	0.385**		−0.81**	2.755**
(AV-REST)^2^			0.012**			0.016*	
FEEDGR-2	0.179	−1.742**	2.384**	−1.304	−19.967	2.625*	−2.335**
FEEDGR-3	2.137**	1.597**	3.769**	1.572**	1.455**	4.308**	−3.423**
LACT-1	0.432*	0.393**		1.299**	−0.994*		
LOCAL-1	−0.946**	−0.458*	−1.133**	–	–	–	–
LOCAL-2	0.238	0.167		–	–	–	–
BREED-HF		–	–	–	–	–	–
INTERCEPT	4.613**	6.576**	40.524**	-17.9213**	2.165	10.731**	– 157**

*AV-ACTIV *average of activity, *AV-HACTIV *average of high activity, *AV-INTAKE* average of feed intake, *AV-RUMIN* average of rumination, *AV-REST* average of rest, *FEEDGR-2* feeding group with milk yield over 11 kg milk per day, *FEEDGR-3* feeding group with milk yield less than 11 kg milk per day, *LACT-1* first lactation, *LOCAL-1* cows in the barn, *LOCAL-2* cows in the pasture, *BREED-HF* Holstein–Friesian breed.

*p < 0.05.

**p < 0.01.

**Table 4:** Criteria of matching quality and prediction for individual datasets.

Criteria Dataset/count	Rate R^2^ Negelkerke	BIC	Hosmer Lemeshow’s p-value	AUC
HF-BS/3735	0.237	0.367	0.801	0.8350
HF/1989	0.267	0.441	0.397	0.8439
BS/1746	**0.375**	**0.256**	**0.949**	**0.9140**
HF-CO/1224	0.321	0.461	0.395	0.8607
HF-PA/765	0.217	0.409	0.659	0.8252
BS-CO/1032	**0.297**	**0.234**	**0.972**	**0.8930**
BS-PA/714	**0.437**	**0.323**	**0.894**	**0.9270**

*BIC* Bayesian information criterion, *AUC* area under curve.

Significant values are in bold.

Only in model related to BS-PA set, extending average time of feed intake, had statistically significant effect i on diagnosing mastitis, in the remaining cases this effectwas negative. In majority of analysed models, variables related to motion activity or ruminating had a significant effects on detecting mastitis, for which a high rate of correlation was observed. Also in this case it is difficult to explicitly determine common direction of influence of those variables on all models. However, it seems that increase in motion activity results in lower chances of diagnosing mastitis. Similarly, longer period of motion inactivity, in case of the analysed models has different direction of influence. Those seemingly contradictory observations that are signalled above can result from accumulative activity of variables in individual models. It makes it hard to formulate general conclusions with reference to individual variables.

Among quality variables, feeding group variable had a substantial impact on probability of developing mastitis. In all the analysed models except the model based on BS-PA set, cows from group 3 were at higher risk of developing mastitis compared with cows from group 1 (group 1 was a level of reference). For BS-PA model, a reverse relationship was observed, a chance of developing mastitis was substantially decreased (−3423). Influence of feeding group 2 on developing mastitis is less explicit from the perspective of the analysed models. In turn, being in the first lactation had a substantial impact, combined with other variables, on probability of developing mastitis. On the basis of Table 3, it can be observed that first lactation variable, was deemed essential and added only to models concerning HF cows, and had a diversified direction of influence.

Different measurements of adequacy evaluation of models used in seven datasets were applied to the hierarchical structure (Table 4). Conformity of conclusions is the main argument to claim that the analyses were done correctly. In general, quantities of criteria that were obtained for all datasets, can be deemed satisfactory. It is true that ranks of matching according to various criteria are not identical, but still it is possible to claim that models for BS breed can be seen as better matched (values in grey). On the other hand, regression models describing HF and HF-PA sets of cows were matched to a lesser degree. It seems that low frequency of mastitis could substantially influence the model’s adequacy in case of cows staying on grazing land. Cows with symptoms of mastitis are usually transported to barn. Model matching is not only dependent on a number of observations. Criteria values for the whole material (all together, 3735 observations of both breeds), correspond with this thesis/argument.

Table 5 shows classification effects with logistic regression and cut-out point of ${\pi }_{0}$= 0.079, determined with Juden’s index for trial test for HF-BF set and for validation in the same dataset (${\pi }_{0}$= 0.067).

**Table 5:** Classification of cases with logistic regression for HF-BS set.

	Training dataset			Validation dataset		
	Classified as sick	Classified as healthy	Sensitivity	Classified as sick	Classified as healthy	Sensitivity
Observed (sick)	154	55	74	159	50	76
Observed (healthy)	707	2819	80	840	2686	76

Accuracy in this model with cut-out points determined this way is ACC = 0.7960, sensitivity SE = 0.7368, and specificity SP = 0,7995, for validation respectively ACC = 0.7617, sensitivity SE = 0.7608, and specificity SP = 0.7618.

## Discussion

Data obtained directly from the system during the research and then enriched with additional information, were not a comfortable source of data allowing building up neural and statistical models because of their number (about 1 million records), their diversity and the fact that they were made available in the form of text file. Because of that, the data migration process was carried out, to structures embedded on SQL level Server 2017. Using possibilities of T-SQL language, actions were taken to improve data quality and indirect structure was created, which included aggregates obtained with grouping questions based on built-in statistical functions. Grouping was carried out based on the column cow’s identifier and date. This additional relation together with merging substantially speeded up realization of questions, which were used to create sets used in the process of building up the aforementioned models. The process of generating datasets was not trivial because of the adopted research assumption. One of the aims was to determine a period of mastitis occurrence. The system contained only information about the day of diagnosing the disease. However, in the process of creating sets, a rule was adopted that three days before disease detection and two days after disease detection were to be considered as morbidity. The recognized periods in the form of unique dates, were one of the essential criteria in selection, during the process of creating datasets concerning sick animals and healthy animals. Another limitation in generating those sets included and taking into consideration only data coming from days when temperature and humidity index (THI) was lower than 72. According to research by Armstrong^18^ and survey article by Polsky and Keyserlingk^19^, a comfort threshold of THI for cows is 72. Additional conditions were adopted in the process of creating datasets concerning healthy animals. Only cows that did not show any morbidity symptoms during the whole period of research were assigned to this group. Cows suffering from other, concomitant diseases are characterized by substantial changes in behaviour in comparison with healthy cows a lot of days before diagnosis^14,20^, for this reason, such cows were deleted from the dataset.

**Figure 1:**
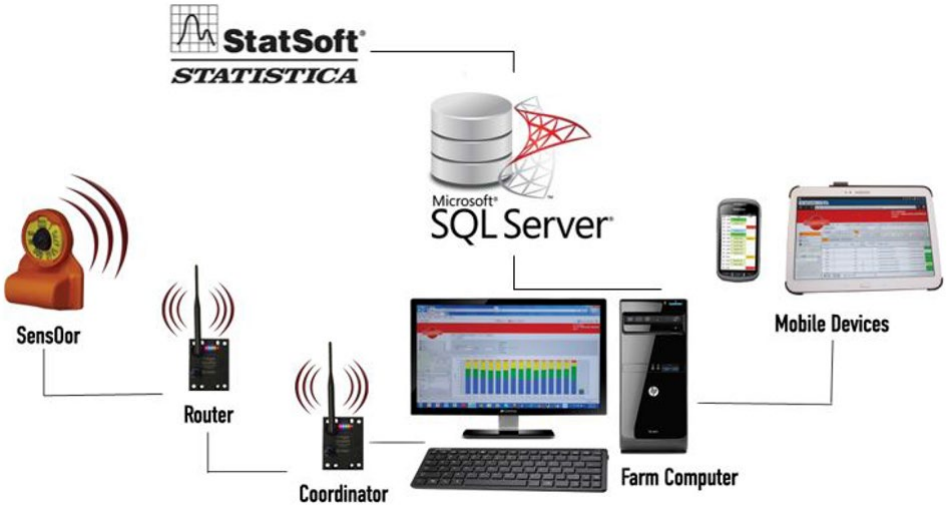
View of the system’s structure, transmission routes and data analysis.

Before the research, the system shown in Fig. 1 was calibrated to adapt its sensitivity to specificity of the herd. It was made on the basis of observation of a selected cow’s behaviour every minute for the period of one hour and then it was compared with data coming from the system. After 56 h of observations, including different cows, the following coefficients/rates of compliance determination of records in surveys and readings from the systems were obtained: taking up feed R^2^ = 0.86, ruminating R^2^ = 0.97, rest R^2^ = 0.94^21^. It was agreed that the system classifies individual types of cows’ behaviours in a sufficient way. The process of selecting optimal set of clarifying variables for a given model (model 1), was carried out with strategy of reverse steps, starting from model with all measured variables, which described behaviour of cows with concomitant quality variables. In another steps, irrelevant variables were eliminated. A detailed description of the procedure that was given by Hosmer and Lemeshow^22^. The idea of isolating learning test and trial test was abandoned because of small number of infection cases in the analysed sets. In order to evaluate a degree of model matching, a valuation technique was adopted based on v-repeated cross-testing^23^. Due to this strategy, there was possibility to evaluate quality of model matching without necessity of creating trial test.

Verification of the model included evaluating of: importance of variables and groups of variables, goodness of fit of the model with observed data and quality of prediction. The obtained error values, despite begin satisfied, made the authors build up another model on the basis of the previously signalled subsets, eliminating the influence of some factors (Table 1). It should be emphasized that in case of the remaining sets, MLP network turned out to be the best network. Low and similar RMS error values for training sets, validation sets and testing sets, prove that the generated ANNs have good generalization features and in turn it means good classification capabilities. Therefore both small RMS error and AUC rate values indicate that the proposed neural models used to detect mastitis on the basis of the accepted input variables are characterized by proper/adequate capability of knowledge generalization. They also indicate that the actions that were taken in the form of creating subsets as way of eliminating influence of some factor did not have substantial impact on changes during the process of classification improvement. Both RMS and AUC values showed negligible fluctuations (Table 2). The key variables in the process of ANN concerning sets HF-BS, HF, BS (including animals of both breeds and animal from just one breed), are quality variables, to which we include feeding group and location to a lesser degree. Quantitative variables extracted from the system with 3D motion sensors are equally important. These are quantities (estimators) connected with feed condition and one of the activity forms separate for one BS model.

The remaining models concerning subsets, where impact of breed and incomplete way of location (animals staying partially in barn were assigned to grazing land group) was consciously eliminated, had its root cause in limited number. From the perspective of gravity/importance of individual variables, some similarity was visible in connection with the previously analysed group. In this case, quality variable „feeding group” turned out to be still significant, and there was another quality variable „first lactation”, which kind of „took the floor”. In terms of gravity/importance, they are mostly separated from quantitative values coming from the system monitoring condition of animals’ activity. However, it is not easy to notice any regularity out of 12 estimators concerning animals’ conditions.

For the seven detailed datasets, the remaining five values related to activity were initially taken into consideration in further actions. However, the estimated coefficient of variance inflation factor for single variables counted in a couple of hundreds, clearly signalled the need for limiting the number of variables. Reduction of the number of continuous clarifying variables to four variables, substantially improved VIF coefficient, which were below 2.5. In the process of selecting reduced number of variables for single data sets, the authors took advantage of additional reverse step procedure. Selection of completely different set of variables caused model matching with similar characteristics, which is justified in correlation between variables (Table 3).

Analysing the results in Table 3, it can be observed that continuous variables coming from the system using 3D motion sensors had a substantial impact on evaluating the probability of developing mastitis. Variable, which turned out to be essential in almost all models, was time of activity related to taking feed, with the exception of HF-CO group. In this case, rest was an essential variable, which, as was already mentioned, was correlated with the eating variable. Despite the fact that this variable did not always show linear relationship with logarithm of odds, after including it as the second degree variable, it had a substantial influence on improving quality of model matching. It is worth noting the fact that for HF-BS model, built up on the basis of the set including both breeds, breed was not deemed essential as a variable used to recognize symptoms of mastitis. Different observations concern location variable, which has a substantial modifying effect. It refers to models marked HF-BS, HF and BS. To sum up, the presented neural models and models of logistic regression show similar capability of classification for healthy animals and for animals with mastitis. It should be added that the above models were built upon the basis of the processed data coming from the information system cooperating with 3D motion sensors and were enriched with quality variables. Similar AUC values, which are common classification quality rates for both models, prove the above assumption. It also should be noted that a similar character of ROC curve was obtained for all models. In both approaches the same quality variables concerning feeding group and location had a substantial influence on models. Being primiparous had a substantial importance only in relation to models built up on sets concerning HF cows. It was observed that breed had different impacts on detecting mastitis, with some surprise. This variable was important for neural model, but it did not appear/occur in logistic regression. HF-BS set was the basis for building up both models.

In case of essential continuous variables, describing animal’s activity, it was very difficult to observe some regularity in both variants for all models, which is the result of the correlation that was found. However, we observe a substantial influence of time spent on feed taking on detecting mastitis in all neural models and in all models of logistic regression. All the remaining continuous variables are characterized by random rank from the perspective of neural models that were obtained. However, in logistic regression one cannot find a simultaneous influence of all continuous variables. Their sets, essential for individual models, are diversified/different. Currently, in the specialist literature, many publications describe the possibility of detecting mastitis by measuring resistance in milk or measuring the content of somatic cells in milk^24,25^. Cavero et al.^26–28^ in those research, they used three different approaches for the same data, which allowed detecting mastitis on the basis of somatic cells in milk. They found the following SE and SP values respectively for models fuzzy logic, locally weighted polynomial regression and neural networks: SE 83%, 88% and 79% while SP: 76%, 67% and 61%. Jensen et al.^29^ on the basis of data coming from many different sensors (among other things: milk yield, milk conductivity, fat and milk protein content, number of somatic cells in body mass), created an algorithm, which allows to process and categorize data in real time. Due to it, it was possible to match models on levels: AUC 0.81 and (SP 81% and SE 80%). Post et al.^30^, who used in their research on cow classification data coming from milking room (volume of milk, conductivity, milk flow, somatic cell count) and data related to feed intake and general animal activity, which was measured with pedometers, obtained similar results. Among numerous models that were tested, the best matching was obtained for Extra Trees Classifier AUC 0.79. In both research with models that were selected, the highest ranks got the models with information about somatic cells.

Referring to the aforementioned works, it is currently difficult to find research that uses such a broad spectrum of data coming from motion detectors, describing behaviour of cows, in their mathematical models to detect mastitis. However, many works indicate changes in the behaviour of cows suffering from mastitis, which—as it was pointed out—can be the basis for classifying healthy and unhealthy/sick animals.

## Conclusion

Generally, the applied neural and logistic regression models were satisfying. Small errors in the MLP neural models reflect their good generalization properties. While measurements of matching and prediction of regression models do not form the basis for their rejection. It is worth highlighting that in both approaches AUC with identical location of ROC curve (exceed 0.8).

The applied models indicated substantial differences in quality variables from the perspective of classification of animals suffering from mastitis: feeding group and location as well as continuous variables obtained from the information system determining period of time of various forms of animal activity. The diversified input of continuous variables in both types of models has its origin in the correlation between variables and imperfections of the information system cooperating with 3D motion sensors, despite their calibration. An attempt that was made to eliminate the impact of some variables by reducing database in the process of constructing both types of models did not substantially improve their classification. Nevertheless, the models, by their reproduction in the information structures, within the frames of a system based on 3D motions sensors or on the basis of the new system can be helpful in the process of detection of mastitis.

## Methods

### Research material and management

The research was conducted on the Juchowo ecological farm located in the West Pomerania Province in Poland. Brown Swiss cows (BS) and Holstein Friesian cows (HF) are housed on the farm with free-stall boxes (for cows in lactation phase) and free-stall boxes with deep bedding (for cows in dry period). Average productivity of the herd was 6500 kg of milk in standard lactation. Cows were milked once a day at different intervals.

In the winter, the cows were fed with hay ad libitum and supplementation in the form of feed concentrates. During grazing season, if it was possible, animals were grazing green fodder growing on grazing land, and in barn animals received supplementation in the form of feed concentrates. The amount of feed concentrates was dependent on lactation stage (from 6 kg at the beginning of lactation to 1 kg at the end of lactation of feed concentrate per cow per day). Food concentrate was divided into two portions and was given to animals after morning and afternoon milking. The experiment started in August 2016. 118 cows were randomly selected (64 HF milk cattle and 54 BF milk cattle). The recorded animals varied according to age and lactation stage.

### Registration of behaviour and production traits

CowManager Sensor, CowManager B.V., were attached to the recorded animals. The sensors were installed in accordance with the producer’s recommendations, on the left auricle. The sensors measured cows’ activity with acceleration measurement registered by accelerometer in three dimensions (3D). Next the data were transferred to router and then to servers belonging to the system provider, where the algorithm classified data to individual activities (taking up feed, ruminating, rest, low physical activity, high physical activity). The behavioural records were grouped in packages corresponding to consecutive hours. Data packets (hours of measurements) included cumulative number of minutes spent by cow on a given activity. The data were combined with information about mastitis (51 cases), which was detected by veterinary surgeon or by taking culture from a cow suffering from udder inflammation. As a result of the experiment that was carried out, more than 960 thousands of records were collected, which were used to draw up neural and regression models, which allowed detection of inflammation of udder.

Implementation of criterion concerning feeding groups (lactation groups), which was reduced to three groups. This division depends on a daily milk production. Cows, which were 5 days after calving, were assigned to the first group. The daily effectiveness of milk production went down to about 20 kg. After this time, animals were assigned to the second feeding group. The daily effectiveness of milk production in this group went down to about 11 kg. Cows producing less than 11 kg of milk per day were assigned to the third group. On top of that, cows before calving, which were supposed to be dried, were also assigned to the last group. Data modification was also carried out, which involved giving some of the factors numerical values. Numerical and quality values used to build up the models included:

- status of animal—average and standard deviation;
  - rest,
  - feed intake,
  - ruminating,
  - activity,
  - high activity—heat behaviours and cattle rush
  - computed total activity
- breed;
- feeding group;
- location;
- lactation number;
- health group.

The dates of observations for each animal (with ID) were recorded. A total of 3785 records were included to build up the predictive models of mastitis occurrence under a previously signalled restrictions. The process of creating the aforementioned sets including the aforesaid factors was carried out with T-SQL language structures such as named, sub questions and unions, which were finally embedded in the procedures. They can act as elements of the future information system, supporting diagnosis of mastitis in dairy cattle.

ANN as well as the logistic regression models from the perspective of mastitis detection, was realized on the basis of seven datasets (Table 6). The first two datasets included data concerning all animals from a given breed irrespective of their place of location. On the hand, the next four disjunctive datasets included information about individual animals from two different breeds in two different locations (grazing land and barn). In the second case, during the process of creating a set related to the location called grazing land, a rule was adopted to assign only those animals, which stay in this location for a few hours per day or for 24 h. The last analysed set included combined data, where breed and location were added to the models as factors. Quantitative and quality variables adopted in creating models, were signalled previously. In case of qualitative variables such as lactation group and location, three levels were included, but the first group was a dichotomous variable.

**Table 6:** Datasets marking.

Marking	Description
HF-BS^a^	cows of both groups (Polish Friesian Holstein and Brown Swiss) in both locations
HF^a^	Polish Friesian Holstein cows staying in all locations
BS^a^	Brown Swiss staying in all locations
HF-CO	Polish Friesian Holstein cows kept in barn
HF-PA^a^	Polish Friesian Holstein cows staying both on the grazing land or in barn
BS-CO^a^	Brown Swiss cows kept in barn
BS-PA^a^	Brown Swiss cows staying both on grazing land and in barn

Marked sets ^a^in the process of building up logistic regression models, some variables had the form of quadratic function.

## Methods of data analysis

### Neural modeling

Creating neural models involves searching for a type of network that is adequate for the set goals. On this basis it is possible to test various types of neural networks on Statistica level such as: line network, probabilistic neural network (PNN), generalized regression neural network (GRRN) network, MLP network and networks with radial base functions (RBF). The structure of sets that were taken into consideration consisted of input variables and 1 nominal output variable. Creating learning sets is inseparably connected with generating the remaining sets; validation set and testing set. It is carried out according to standard schema 2:1:1, which determines the number of subsets in relation to the whole. The testing subset does not take part in generating the classification model, which is the model the authors deal with. Creating training set becomes the basis for learning process, where we try to minimize learning error by using various types of learning algorithms. Another step is to validate quality of a given neural model, as a result of which we obtain appropriate quality measurements. While the last verification are parameters obtained on testing set. Root Mean Square (RMS) error is a standard measurement of classification accuracy of the generated ANN. This measurement is defined as accumulative error made by network on datasets. It is calculated according to the following formula:

$$RMS=\sqrt{\frac{\sum_{i=1}^{n}{({y}_{i}-{z}_{i})}^{2}}{n}}$$

where: n is the number of all cases in learning sets, y_i_ is the ith empirical value, z_i_ is the ith values determined by ANN.

Small values of RMS error can signify that MLP neural types are characterized by appropriate capability of generalizing knowledge in classification issues.

### Logistic regression

Another alternative approach used in this work is logistic regression. Dependent variable, similar to output variable in neural models had dichotomous character: 1 (cow with mastitis) versus 0 (healthy cow). It is assumed that we observe *n* of independent pairs of forms $\left({{\varvec{x}}}_{i},{y}_{i}\right)$, $i=1,\ldots ,n$, where ${{\varvec{x}}}_{i}^{{\prime}}=\left({x}_{0i},{x}_{1i},\ldots ,{x}_{pi}\right)$ and ${x}_{0i}=1$, means vector $p+1$ of determined variable values for ith of this unit, and ${y}_{i}=0 \mathrm{or} 1$ means, the aforementioned realization of random variable ${Y}_{i}$ .

In the model of logistic regression, the formula of conditional probability is determined:

$$P\left({Y}_{i}=1/{{\varvec{x}}}_{i}\right)=\pi \left({{\varvec{x}}}_{i}\right)=\frac{{e}^{{{\varvec{x}}}_{i}^{{{\prime}}}{\varvec{\beta}}}}{1+{e}^{{{\varvec{x}}}_{i}^{{{\prime}}}{\varvec{\beta}}}}.$$

After processing and finding the logarithm of the above formula we obtain:

$$logitP\left({Y}_{i}=1/{{\varvec{x}}}_{i}\right)=ln\frac{P\left({Y}_{i}=1/{{\varvec{x}}}_{i}\right)}{1-P\left({Y}_{i}=1/{{\varvec{x}}}_{i}\right)}={{\varvec{x}}}_{i}^{{{\prime}}}{\varvec{\beta}}.$$

The left side of the equation is called an odd of mastitis occurrence. In the processed model, we estimate the logarithm of chance and assume that it depends on a linear way of clarifying variables. Vector of parameters $\beta$ in the model was estimated using the Maximum Likelihood method adopted by Hosmer et al.^31^.

In the case of each independent variable that was analysed, a possible relation with the dependent variable was checked, and then a model with single variable was determined, and verification of its significance was carried out. Because of determinants of logistic regression, as part of initial analysis of data for continuous independent variables, a correlation matrix was determined. In order to diagnose the occurrence of collinearity, a variation inflation factor (VIF) was determined, which should not exceed 2.5^32^.

The significance of variables in the model were examined by likelihood-ratio test (LR) and incremental chi-square statistic test and Wald’s test^33^. Adequacy of model was verified with Hosmer and Lemeshow test^22^, which compares distribution of expected numerical strength with the observed numerical strengths in groups. Pseudo R^2^ Nagelkerke^34^ was also used to select the best model. It is based on the likelihood function and describes an improvement of predictions in the model relative to model with only intercept. Pseudo R^2^ measurements are much lower than classical determination coefficient R^2^ in regression models, its typical value is 0.2 to 0.5^22^. Measurement value of Pseudo R^2^ similar to classical R^2^ increases if we add another variables to model, that is why verification of the degree of model matching was also carried out with Bayesian Information Criteria^35^. On the basis of SE and 1-SP values, a ROC (Receiver Operating Characteristic) curve was constructed for all possible cut-out points. ROC curve was used as a tool to evaluate and compare classification models between each other. Area under ROC curve marked as AUC, can be treated as measurement of discriminative quality of a given model^36^. AUC values ranged from 0 to 1. AUC value above 0.5 indicates classification better than random classification. In the process of selecting the cut-out point for classification, Jouden index J = SE + SP − 1 was used. The optimal point of cutting-out is when the Jouden index takes maximum value Jouden 1950. Apart from regression coefficients, odds ratio was also estimated.

An assumption of linear relationship between logarithms of odds and independent variables with logarithm likelihood test, which compares models with a given variable and quadratic variables, was checked.

Another stage of model verification is an evaluation of predictive ability of model With the use of the model we predict the probability of success if this probability is higher than the set value ${\pi }_{0}$, called cut-out point we assume that mastitis occurred $\left(\hat{y}=1\right)$, otherwise mastitis did not occur $\left(\hat{y}=0\right)$. In logistic model it is assumed that cut-out point is usually 0.5.

With low frequency of occurrence of a given case, a lower value of ${\pi }_{0}$ can be adopted, on the basis of the observed occurrence^37^.

On the basis of the predicted values, a classification matrix was created, in which number of properly and improperly classified cases was given (TP—true positive) and (TN—true negative) vs. (FP—false positve) and (FN—false negative).

Measurements of quality classification were also determined, which are commonly used in diagnostic models, namely accuracy, sensitivity and specificity.

$$\mathrm{Sensitivity}: SE=\frac{TP}{TP+FN} \quad \mathrm{ Specificity}:SP=\frac{TN}{TN+FP}$$

$$\mathrm{Accuracy}: ACC=\frac{TP+TN}{TP+TN+FP+FN}=\frac{TP+TN}{N}$$

These calculations (for both methods) were performed in Statistica package programs (Statistica version 13.3).

### Ethics approval and consent to participate

The authors confirm that the study was carried out in compliance with the ARRIVE guidelines. The Second Local Ethics Committee for Animal Experimentation SGGW of the Ministry of Science and Higher Education (Poland) reviewed and approved all procedures. The consent of the local ethical committee WAW 2/70/2016 dated 12.16.2016. All cows were handled in accordance with the regulations of the Polish Council on Animal Care, and the Warsaw University of Life Sciences Care Committee reviewed and approved the experiment and all procedures carried out in the study.

## Data Availability

All data generated or analysed during the study are included in this published article. The datasets used and/or analysed in the current study are available from the corresponding author on reasonable request.
